# Editorial: Gut permeability-related endotoxemia and cardiovascular disease: A new clinical challenge

**DOI:** 10.3389/fcvm.2023.1118625

**Published:** 2023-03-21

**Authors:** Francesco Violi, Cristina Nocella

**Affiliations:** ^1^ Department of Clinical Internal, Anaesthesiologic and Cardiovascular Sciences, Sapienza University of Rome, Rome, Italy; ^2^ Mediterranea Cardiocentro-Napoli, Naples, Italy

**Keywords:** gut microbiota, gut permeability, thrombosis, cardiovasclar disease, LPS (lipopolysaccharide)

**Editorial on the Research Topic** Gut permeability-related endotoxemia and cardiovascular disease: A new clinical challenge

## Introduction

Emerging evidence indicates that gut dysbiosis is a risk factor for cardiovascular disease (CVD) in type 1 diabetes mellitus (1), obesity (2) or hypertension (3). Gut dysbiosis is characterized by an overgrowth of gut bacteria that ultimately leads to enhanced intestinal barrier permeability with bacteria or bacteria products such as lipopolysaccharide (LPS) translocation into the systemic circulation (4). Other triggers of dysbiosis-related endotoxemia include aging, and alcohol abuse (4); also, several conditions such as non-septic pneumonia (5–7), acute phase of myocardial infarction, experimental models of intestinal anoxia (8, 9) and systemic inflammation-associated overproduction of pro-inflammatory cytokines, such as interferon-*γ* and tumour necrosis factor alpha (7), increase gut permeability and ensuing low-grade endotoxaemia. LPS is the main component of the outer leaflet of gut Gram-negative bacteria and is composed of glycolipid containing carbohydrates and a lipid A portion (4). Low, non-pathologic levels of LPS may be detected in human circulation; LPS values >20 pg/ml but 2-fold lower compared to septic patients, have been recently indicated as reflecting low-grade endotoxemia (10). LPS levels may also change physiologically after food intake as LPS is embedded in freshly synthesized chylomicrons and, thereby, reaches the systemic circulation after crossing the intestinal barrier (11).

The human body encompasses local and systemic mechanisms to counteract LPS translocation or to detoxify it. Local defence mechanisms include the intestinal mucus that separates gut microbiota from the intestinal barrier consisting of epithelial cells, that are held together by adhesion proteins such as tight junction (TJ) proteins, adherence junctions proteins (cadherins and catenins), gap junction (connexin) proteins and desmosomes (desmoglein and desmocollins). Just below the epithelial barrier, the gut vascular barrier is also implicated in modulating bacteria translocation into the portal vein (12). Two sequential major LPS detoxification processes occur at the level of intestinal or liver cells in the case of damage to the intestinal barrier: (1) production and secretion, by intestinal epithelial cells, of the High Density Lipoprotein (HDL) 3 that exerts an anti-inflammatory activity by binding LPS-binding protein (LBP) so failing LPS recognition by its receptor TLR4 at the level of liver macrophages; (2) synthesis of intestinal alkaline phosphatase, which removes the lipid A phosphates of LPS giving formation of monophosphoryl-LPS, that, differently from the entire molecule, acts as TLR4 antagonist and (3) the liver acyloxyacylhydrolase, which deacylated critical fatty acid residues for LPS recognition (4).

**Figure 1:**
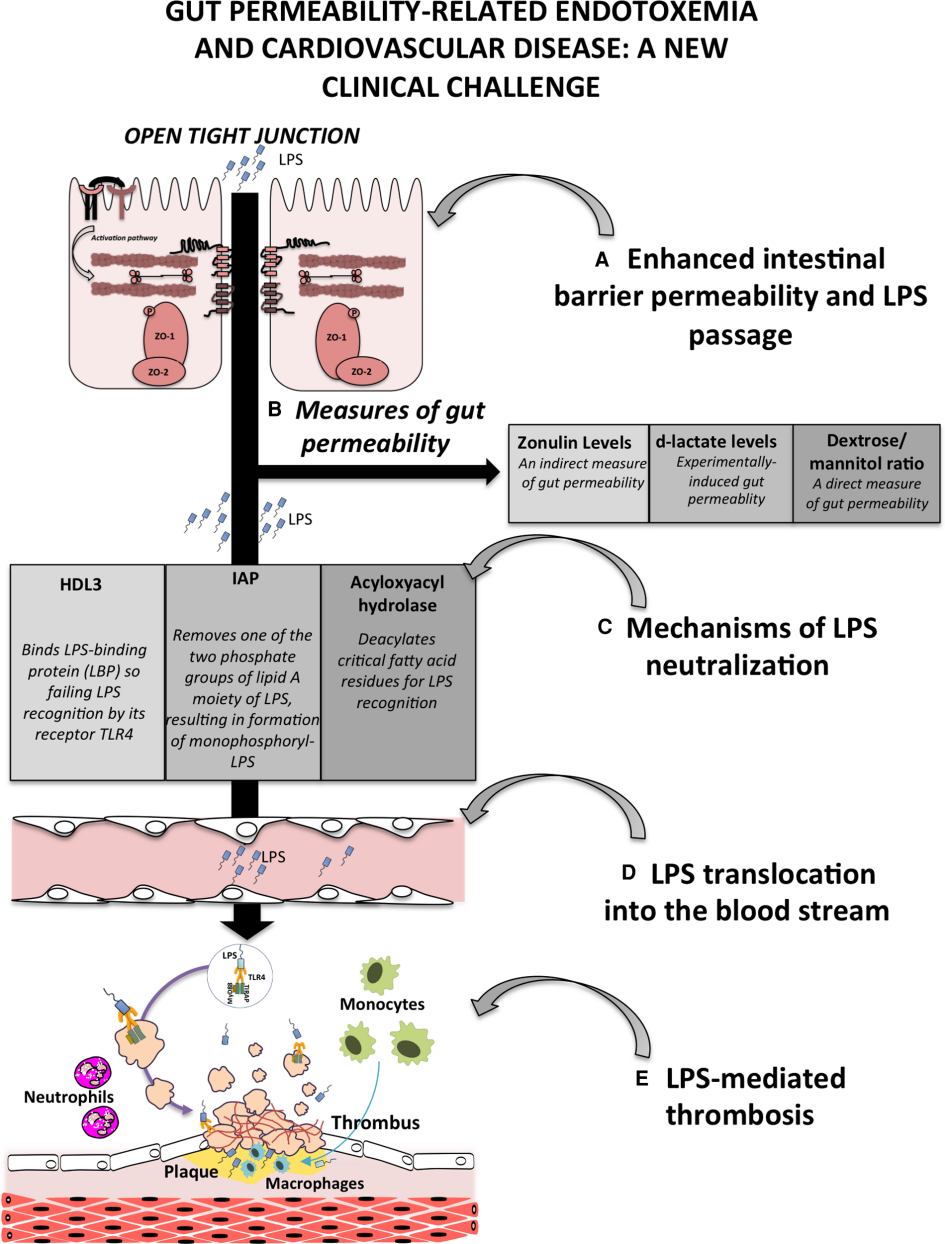
Schematic representation of gut permeability-related endotoxemia and cardiovascular disease. (**A**) Enhanced intestinal barrier permeability is a consequence of gut dysbiosis that, by the down-regulation of intestinal adhesive proteins, leads to the translocation of bacteria or bacteria products, such as lipopolysaccharide (LPS), into the systemic circulation; (**B**) gut permeability can be measured by several approaches including serum levels of zonulin, the analysis of d-lactate levels in the blood and the measurement of urinary excretion of dextrose and mannitol; (**C**) at the level of intestinal or liver cells, several processes occur to detoxify LPS such the production and secretion of the lipoprotein HDL3, the activity of the intestinal alkaline phosphatase (IAP), which dephosphorylates LPS, and the liver acyloxyacylhydrolase, which deacylated critical fatty acid residues for LPS recognition; (**D**) failure of mechanisms to detoxify LPS favours its translocation into the bloodstream, and finally (**E**) the atherothrombosis mediated by several cells including platelets, endothelial cells, monocytes, macrophages and neutrophils.

There are several methodology to measure gut permeability in human, the simplest ones relate to serum measurement of molecules that provide indirect information such analysis of zonulin or D-lactate. Zonulin is a paracrine protein, with a molecular mass of 47-kDa, released by several cell lines, including intestinal cells, after exposure to gliadin-specific peptides or bacteria (13). Activation of the zonulin pathway induces, by protein kinase C-mediated cytoskeleton reorganisation, the disengagement of the protein ZO-1 from the tight junctional complex followed by an increase in intestinal permeability (14). Studies on type 2 diabetes, obesity (15, 16) or acute or chronic CVD (17) patients revealed an increase in serum levels of zonulin coincidentally with elevated levels of LPS. Analysis of serum D-lactate is another alternative because, if increased, it may reflect an efflux of bacteria or their products into circulation consequent to mucosal injury or experimentally-induced gut injury (18, 19). However, a better approach for analysing gut barrier dysfunction is the direct analysis of urinary excretion of dextrose and mannitol after oral ingestion of the mixture (20); however, such an analysis is cumbersome and requires specific expertise (21). Change of gut barrier functionality with enhanced intestinal permeability is *conditio sine qua non* to observe increased LPS translocation into the systemic circulation with ensuing endotoxemia (Figure 1). The close relationship between gut dysbiosis-related gut permeability and endotoxemia is supported by an interventional study in animals treated with a large spectrum of antibiotics showing reduced systemic levels of LPS coincidentally with the improvement of gut barrier dysfunctionality (22). Analysis of gut dysfunctionality has been assessed in different clinical settings including patients at risk or with coronary heart disease (CHD), in whom LPS has been shown to localize within the atherosclerotic plaque in close association with activated macrophages and in coronary thrombi suggesting that LPS possess pro-atherosclerotic and prothrombotic properties (4). Accordingly, prospective studies documented that LPS blood levels are predictive of atherosclerotic plaque progression and cardiovascular events such as myocardial infarction and stroke (23). Based on these findings, gut dysbiosis-related intestinal barrier permeability with LPS translocation into systemic circulation may represent a novel mechanism favouring atherosclerotic burden and thrombosis in the arterial circulation (Figure 1).

Articles published in this Research Topic reported interesting findings and helped with our further knowledge on this topic. Thus, Long et al. analyzed the current state of gut microbiota scientific research and provided a useful perspective in the field of CHD by bibliometric and visualization software. The authors categorized studies into three key current research hotspots including (1) the mechanism by which gut microbiota metabolites, such as trimethylamine N-oxide (TMAO), short-chain fatty acids (SCFA) or LPS, influence the progression of CHD, (2) microbiota-targeted therapy in CHD management and (3) dietary modification as a possible tool to decelerate the progression of CHD. Moreover, they predict 2 research themes that will play a significant role in the future including the research about novel intervention strategies with natural compounds to modulate gut microbiota and its metabolites in patients with CHD and the study of specific mechanisms of action of gut microbiota affecting CHD.

In the review by Sun et al., the authors discussed the role of various statins affecting the gut microbiota and LDL-C metabolism through the gut-liver axis. After oral administration, statins enter the intestine *via* the upper gastrointestinal tract and affect the abundance of gut microbiota, which in turn modulates microbiota-derived metabolites such as SCFAs, secondary bile acids (SBAs), TMA, and LPS. These metabolites, transported to the liver through the portal venous system, can regulate cholesterol metabolism and LDL-C levels by several signaling pathways in hepatocytes. Even if preclinical studies have shown the positive effect of statins regulating LDL-C by gut-liver axis, limited studies are reported for humans.

Inflammation promotes every stage of atherosclerosis, from initiation to progression and evolution. Vreeken et al. described the role of endothelial PLXNA4, a protein that results downregulated in inflammatory conditions. The authors demonstrated that inflammatory stimuli reduce endothelial PLXNA4 expression resulting in rearranged cytoskeletal structures and cell-cell junctions. These structural changes increased vascular permeability to inflammatory cells, such as monocytes, into the arterial wall favouring atherosclerosis.

Finally, Zhang et al. aimed to verify the association between the serum levels of alkaline phosphatase (ALP) and stroke in a cohort of adults with hypertension. They found that hypertensive adults with higher serum ALP had a significantly higher risk of first total stroke.

## Author contributions

FV: writing—review, and supervision. CN: writing and editing. All authors contributed to the article and approved the submitted version.

## Conflict of interest

The authors declare that the research was conducted in the absence of any commercial or financial relationships that could be construed as a potential conflict of interest.
